# The dwarf neon rainbowfish *Melanotaenia praecox*, a small spiny‐rayed fish with potential as a new Acanthomorpha model fish: I. Fin ray ontogeny and postembryonic staging

**DOI:** 10.1002/dvdy.699

**Published:** 2024-02-07

**Authors:** Kazuhide Miyamoto, Gembu Abe, Koji Tamura

**Affiliations:** ^1^ Department of Ecological Developmental Adaptability Life Sciences, Graduate School of Life Sciences Tohoku University Sendai Japan; ^2^ Division of Developmental Biology, Department of Functional Morphology, Faculty of Medicine, School of Life Science Tottori University Yonago Japan

**Keywords:** Atheriniformes, Melanotaeniidae, procurrent ray, soft ray, staging system

## Abstract

**Background:**

Fish fins with highly variable color patterns and morphologies have many functions. In Actinopterygii, the free parts of fins are supported by “soft rays” and “spiny rays.” Spiny rays have various functions and are extremely modified in some species, but they are lacking in popular model fish such as zebrafish and medaka. Additionally, some model fish with spiny rays are difficult to maintain in ordinary laboratory systems.

**Results:**

Characteristics of the small, spiny‐rayed rainbowfish *Melanotaenia praecox* render it useful as an experimental model species. Neither fish age nor body size correlate well with fin development during postembryonic development in this species. A four‐stage developmental classification is proposed that is based on fin ray development.

**Conclusions:**

*Melanotaenia praecox* is an ideal species to rear in laboratories for developmental studies. Our classification allows for postembryonic staging of this species independent of individual age and body size. Development of each fin ray may be synchronized with dorsal fin development. We discuss the differences in mechanisms regulating soft, spiny, and procurrent ray development.

## Key Findings

- Melanotaenia praecox is an ideal species to rear in laboratories for developmental studies.
- We established a four‐stage developmental classification of stages based on fin‐ray development.
- Postembryonic staging of this species is independent of individual age and body size.
- Development of each fin rays seems synchronized with dorsal fin development.

## 1 INTRODUCTION

Fish fins can be highly variable in color and morphology, and have many functions in, for example, swimming, predator defense, and courtship.^1^, ^2^ In Actinopterygii, the free parts of fins are commonly supported by structures called “fin rays,”^3^ which are further divided into “soft rays,” which are flexible, segmented, and sometimes bifurcated, and “spiny rays,” which are stiff, unsegmented, and fused of two half‐segments (hemitrichia), and which terminate in an acute point.^2^, ^4^, ^5^, ^6^ Spiny rays can function in defense against gape‐limited predators^1^, ^2^, ^6^, ^7^ and also act as a leading edge into an oncoming flow.^1^, ^8^, ^9^ They have evolved independently in several teleost lineages.^2^, ^5^, ^10^

Spiny rays of acanthomorph fishes are called a “true spine,” whereas nonacanthomorph groups such as carps and catfish are independently equipped with another type of spines.^1^, ^2^, ^5^, ^11^ Some acanthomorph species have extremely modified spiny rays. For example, the sucking disc of remoras has “pectinated lamellae,” formed by bilateral extensions of the fin spine base.^12^ Anglerfish have an “illicium,” in which spiny rays are modified into and serve as a fishing apparatus.^13^, ^14^ The diversity in form of spiny rays has attracted considerable ecological, morphological, and evolutionary interest. Thus, it is important to understand the mechanism(s) by which these spiny rays form, enabling their diversification in the Acanthomorpha. To achieve this objective, spiny ray morphogenesis is best investigated using laboratory animals.

While one popular acanthomorph model fish, medaka (*Oryzias latipes*), lacks spiny rays, other species with spiny rays are suitable for studies on developmental biology (e.g., the relatively large cichlid *Astatotilapia burtoni* and smaller clownfish *Amphiprion ocellaris*).^5^, ^15^, ^16^, ^17^ Not only is the skeletal ontogeny and fin ray development of *A. burtoni* well described, with dorsal and anal‐fin spiny rays reported to emerge between 7 and 8 days postfertilization (dpf),^15^ but CRISPR/Cas^18^ and *Tol2* transposon^19^ system methods have also been established, enabling gene and cell manipulation in laboratories. Unfortunately, the large adult size of this species renders it an inappropriate taxon for laboratory studies when housing many mutant and transgenic lines.

Postembryonic ontogeny and fin ray ontogeny have also been described for *A. ocellaris*,^16^ for which spiny rays are located in the pelvic, dorsal, and anal fins, and these structures emerge from 5 to 8 dpf. Methods for microinjection and CRISPR/Cas systems for this species have also been established, enabling the production of mutations.^20^, ^21^ However, the clownfish is marine, has a long generation time (~1.5 years),^22^ and must be maintained in a seawater system, which involves more time and greater effort than for some other freshwater species.^21^

Neither *A. burtoni* nor *A. ocellaris* are easy to maintain in ordinary laboratory systems. For laboratory‐based studies involving acanthomorph taxa, a freshwater species of similar maintaining system size to zebrafish or medaka that breeds easily and prolifically throughout the year, has a short generation time, and possibly has an established genome‐editing system, would be ideal.

**FIGURE 1:**
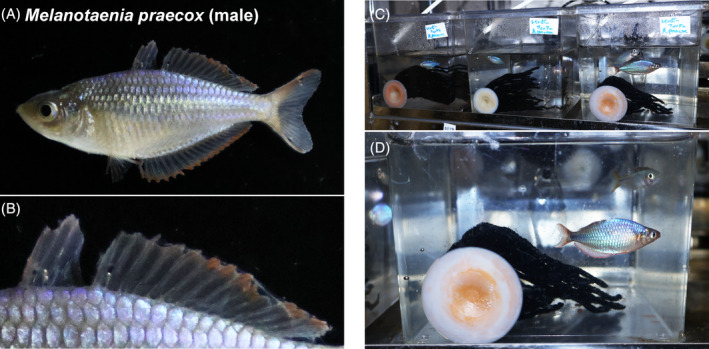
*Melanotaenia praecox* morphology and husbandry. (A) Body, lateral view. (B) First and second dorsal fins. (C,D) Typical tank system. Several tanks (C) with single breeding pairs were established to increase egg‐collection efficiency. (D) Wool mops suction‐clamped to tank wall.

Most rainbowfish (Melanotaeniidae) have spiny rays in their dorsal, anal, pectoral, and pelvic fins.^23^ The dwarf neon rainbowfish *Melanotaenia praecox*, a small freshwater species in the order Atheriniformes native to northern New Guinea (Figure 1),^24^, ^25^ may be suitable as a model fish for studying fin ray ontogeny.^26^, ^27^ Adults of this species are ~3–5 cm in length. While a limited description of the ontogenetic process of the first dorsal‐fin spiny rays exists for some rainbowfish related to *M. praecox*,^23^ to better understand the development of spiny and soft rays requires a greater description of fin ontogeny. This would be facilitated with a schema to stage postembryonic development.

Fin rays of *M. praecox* develop after hatching, but postembryonic fin ray development was hitherto unknown. Accordingly, we describe the postembryonic development of fin morphology in this species. Relationships between fin development and fish age and/or body size (measured using standard length [SL]) are evaluated, and a system to stage postembryonic development is proposed based on fin development. We also examine the relationship between this staging table and the development of all fins. These results improve our understanding of differences in mechanisms regulating soft, spiny, and procurrent ray development in fish species.

## 2 RESULTS

### 2.1 Adult fin skeleton

**FIGURE 2:**
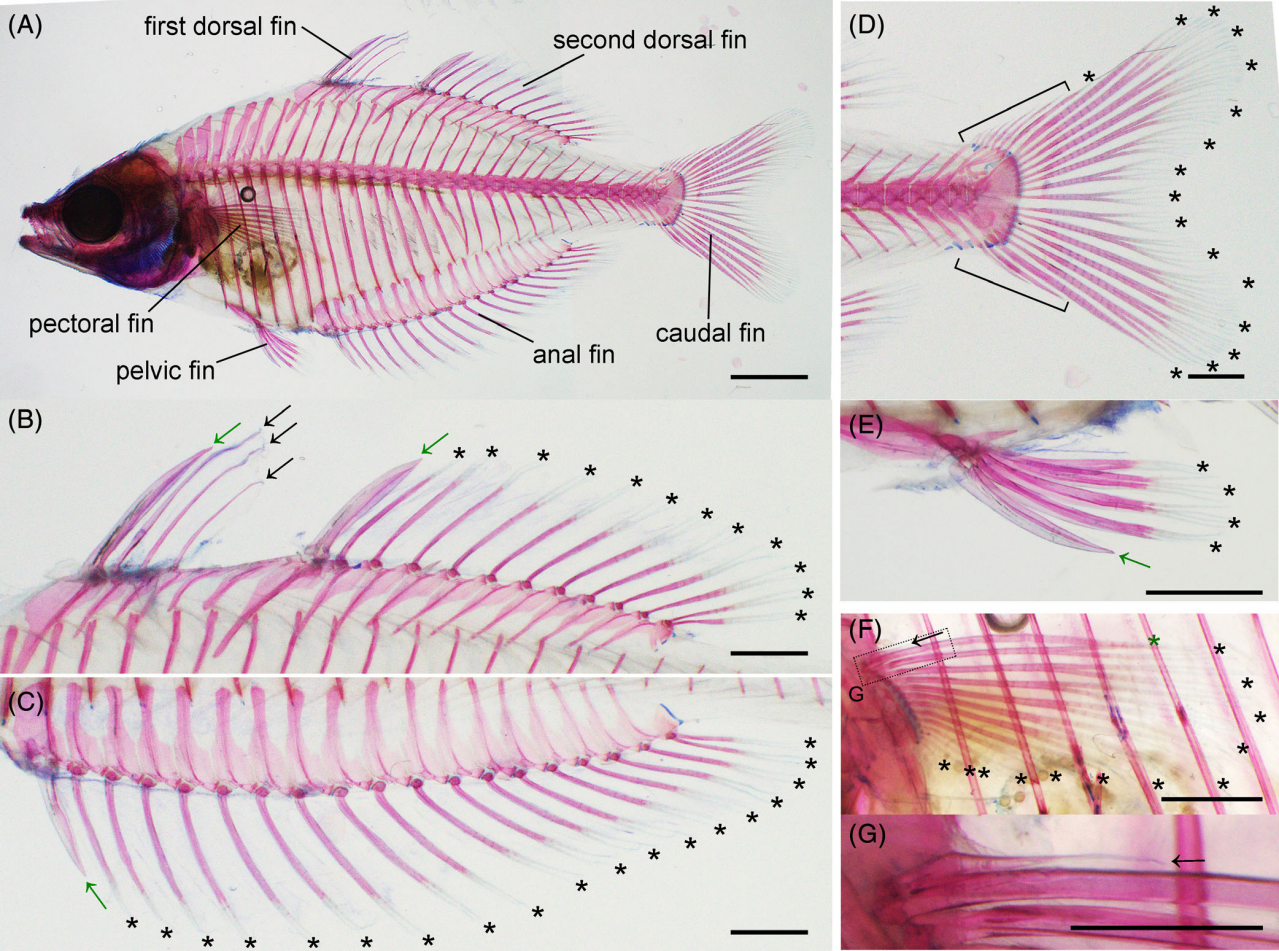
Adult *Melanotaenia praecox* skeleton. (A–G) Alizarin red/Alcian blue stained and cleared skeleton. Lateral view of entire body (A), and magnified views of dorsal (B), anal (C), caudal (D), pelvic (E), and pectoral (F,G) fins. The “spiny‐ray‐like structure” in (G) is a magnified view of (F). Asterisks indicate soft rays. Arrows: black, spiny rays; green, the first spiny ray of each fin. Brackets in (D) indicate sites where procurrent rays existed. Scale bars: (A) 5 mm, (B–F) 2 mm, and (G) 1 mm.

While there are a species description^28^ and reports of adult traits of *M. praecox*,^29^ these descriptions are brief, and the numbers of fin rays in this species are more variable than previously recognized. Thus, we first examined the number of fin rays in adult *M. praecox* fins (*n* = 14, Figure 2 and Table 1). The first dorsal fin (DF) has three to five spiny rays (arrows, Figure 2B). The second DF has 1 or 2 spiny rays (green arrow, Figure 2B) and 11–14 soft rays (asterisks, Figure 2B). The anal fin has 1 spiny ray (green arrow, Figure 2C) and 18–22 soft rays (asterisks, Figure 2C). The caudal fin has 15–18 soft rays (asterisks, Figure 2D) and several unsegmented, nonbifurcated procurrent rays located along the dorsal and ventral fin edges (brackets, Figure 2D; precise counts of procurrent rays were not possible because some detached during staining). The pelvic fin has one spiny ray (green arrow, Figure 2E) and four or five soft rays (asterisks, Figure 2E). The pectoral fin has 1 spiny‐ray‐like structure (arrow, Figure 2F,G) and 11–13 soft rays (black asterisk, Figure 2F). The spiny‐ray‐like structures, which the dorsal and ventral hemitrichial elements fuse into, are unsegmented and elongated along the proximal–distal axis. Although these structures could be classified as “fulcra,” “procurrent rays,” or “spiny rays,”^30^ we regard them as spiny rays. Additionally, we regard the fin ray adjacent to the spiny ray in the pectoral fins (green asterisk, Figure 2F), into which the dorsal and ventral elements fuse, as comprising soft rays because they are segmented. The first spiny rays of the first and second DFs and the anal and pelvic fins are distinctly thick (green arrows, Figure 2B,C,E).

**TABLE 1:** Number of fin rays in adult *Melanotaenia praecox*.

	Spiny rays	Soft rays
First dorsal fin	3 (23%), 4 (38%), 5 (38%)	0 (100%)
Second dorsal fin	1 (100%)	11 (31%), 12 (31%), 13 (23%), 14 (15%)
Anal fin	1 (100%)	18 (14%), 19 (14%), 20 (36%), 21 (29%), 22 (7%)
Caudal fin	0 (100%)	15 (7%), 16 (43%), 17 (43%), 18 (7%)
Pelvic fin	Right: 1 (100%)	Right: 4 (14%), 5 (86%)
	Left: 1 (100%)	Left: 5 (100%)
Pectoral fin	Right: 1 (100%)	Right: 11 (14%), 12 (36%), 13 (50%)
	Left: 1 (100%)	Left: 11 (7%), 12 (50%), 13 (43%)

*Note*: Samples with evidence of ray loss are excluded.

We report that the number of fin rays in the adult fins varies. Previous research has shown that the number of pectoral fin rays in zebrafish also varies, suggesting that this variation is affected by intrinsic genetic and nongenetic factors, such as developmental noise and environmental stimuli.^31^ Thus, the number of fin rays in *M. praecox* might also be affected by genetic and nongenetic factors. The number of soft rays in *M. praecox* paired fins occasionally differs between opposite sides of an individual, as reported for zebrafish,^31^ suggesting that this is also a result of nongenetic factors.

### 2.2 Developmental overview

#### 2.2.1 Growth rate

**FIGURE 3:**
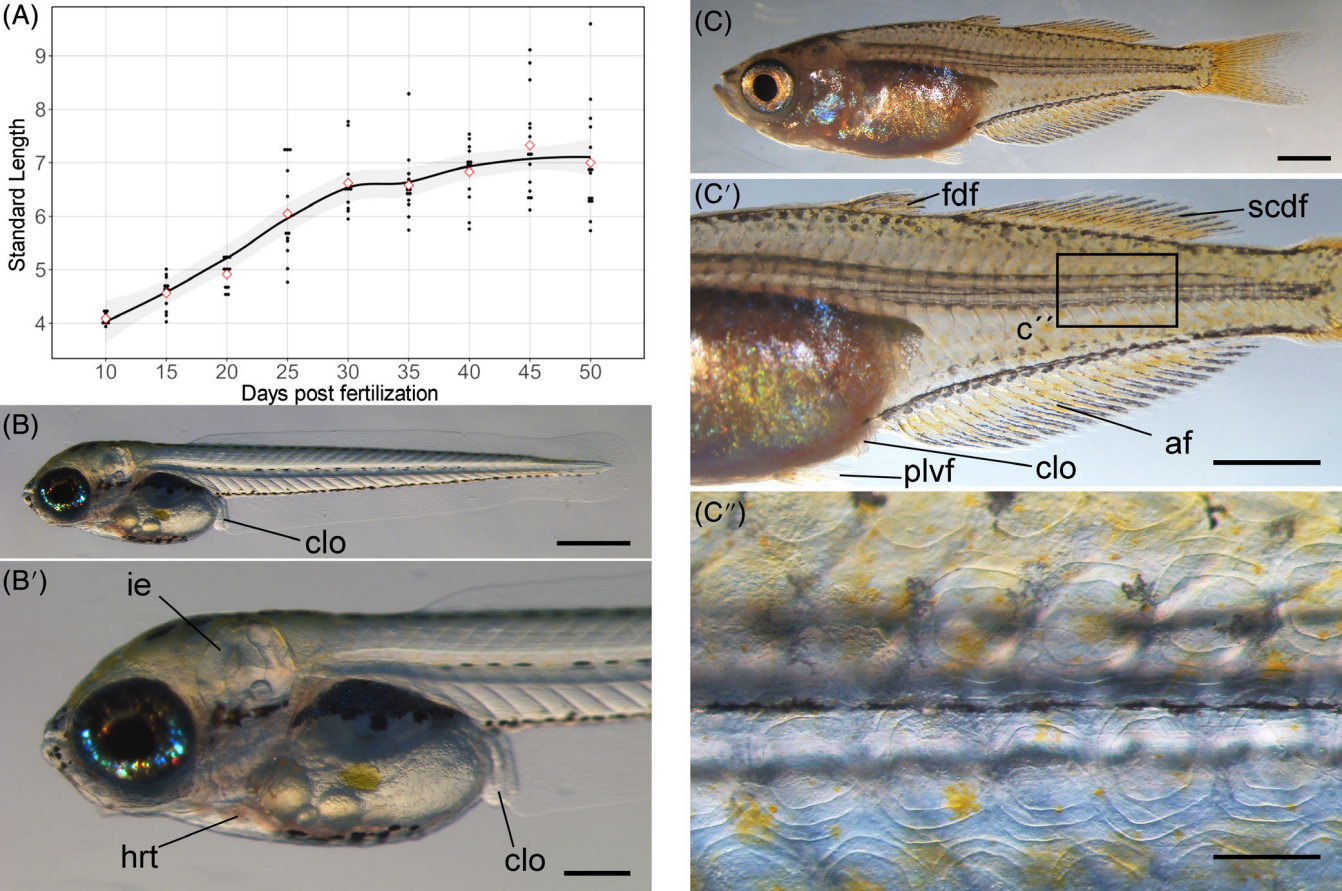
Growth rates and morphology of *Melanotaenia praecox*. (A) Relationship between standard length and days postfertilization; local polynomial regression fit of scatter plots, with 95% confidence intervals indicated in gray. (B,B′) Lateral view, hatched larva (magnified in B′). (C–C″) Lateral views of juvenile (C), with magnified views in (C′), with (C″) being a magnified view of the rectangle in (C′). (C″) shows that scales of juvenile fish are visible under the bright field. af, anal fin; clo, cloaca; fdf, first dorsal fin; hrt, heart; ie, inner ear; scdf, second dorsal fin; plvf, pelvic fin. Scale bars: (B) 500 μm, (B′,C″) 200 μm, and (C,C′) 1 mm.

To examine the growth rate, SL was measured at 5‐day intervals from 5 to 50 dpf (Figure 3A). Because variation in growth rate produced different‐sized individuals for a given age, SL weakly correlates with dpf. Growth rate tends to be influenced by environmental factors such as population density,^32^, ^33^ and SL or qualitative traits have been used as references for developmental stages.^16^, ^32^, ^33^, ^34^ We define postembryonic developmental stages based on fin fold and fin morphology.

#### 2.2.2 Postembryonic stage definition

Embryonic and postembryonic zebrafish and goldfish staging tables define hatching and a protruding mouth to indicate the initial postembryonic stage.^32^, ^33^, ^34^ Although Radael et al.^26^ demonstrated that hatching in *M. praecox* occurs near 5 dpf at 28°C, our larvae hatched from 6 to 8 dpf at 28°C. The larval mouth was wide open and protruded anteriorly just after hatching (Figure 3B,B′). Thus, we define the timing of “just after hatching” as the initiation of the postembryonic stage.

In studies on zebrafish, goldfish, and clownfish,^16^, ^31^, ^32^, ^33^ the initiation of the juvenile stage has been defined by reductions in the larval median fin fold (LMFF), progression of squamation, and change in pigmentation. Squamation in *M. praecox* occurs after a reduction in the LMFF and the end of fin ray formation at ~2 months postfertilization (Figure 3C–C″). There was no dramatic change in pigmentation during development (Figures 3C,C′ and 6). In addition, progression of the squamation is easily observed (Figure 3C″). Thus, we define the “timing of scale emergence” as the initiation of the juvenile stage.

#### 2.2.3 Fin development

**FIGURE 4:**
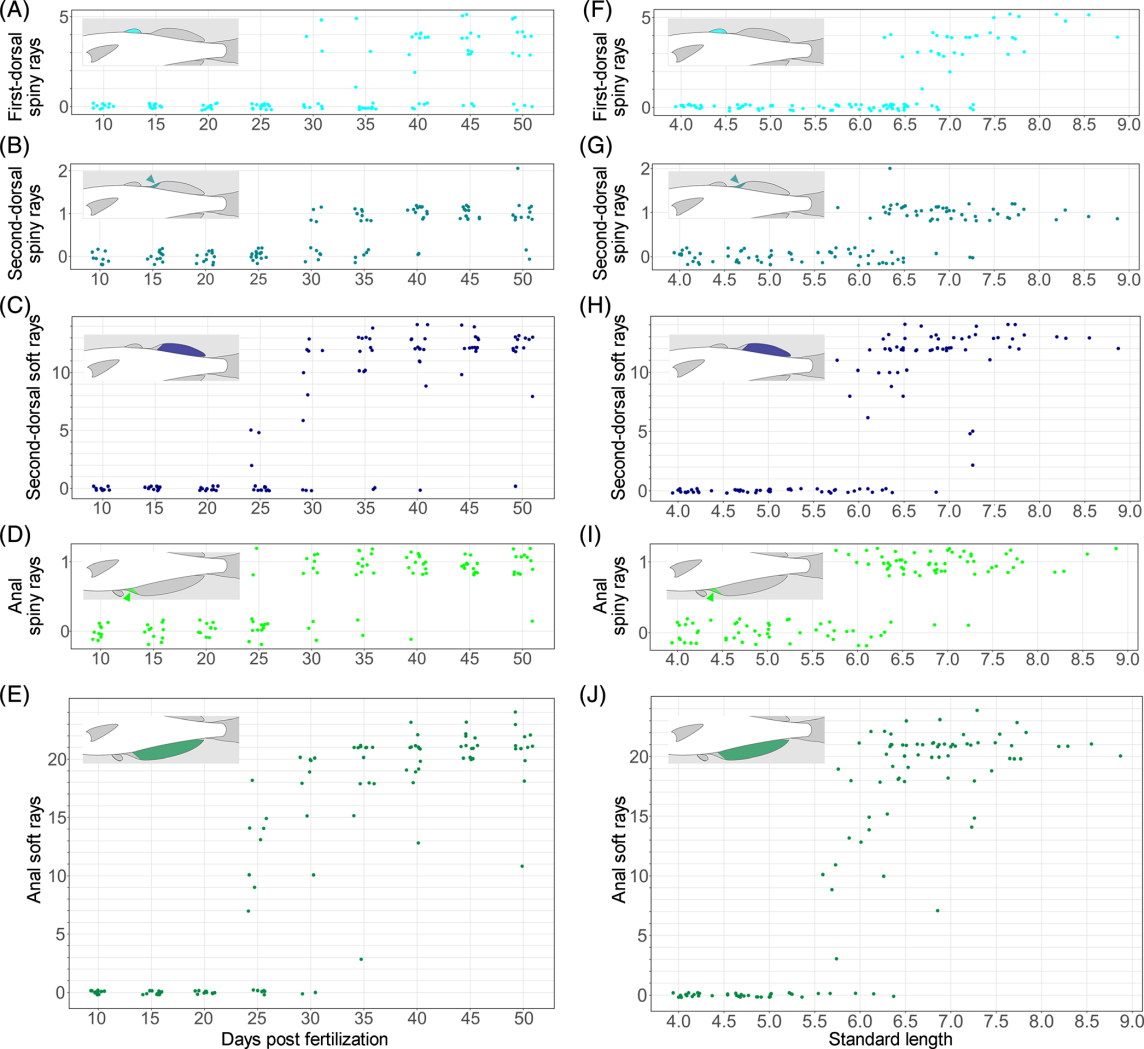
(A–E) Relationships between fin ray (spiny or soft) number and days postfertilization for the first dorsal (A), second dorsal (B,C), and anal (D, E) fins. To reduce data overlap, all points are jittered along *x* and *y* axes. (F–J) Relationships between fin ray number and standard length in first dorsal (F), second dorsal (G,H), and anal (I,J) fins. To reduce data overlap, all points are jittered along the *y* axis. Schematic drawings in each graph (A–J) indicate the position of each type of fin ray. Arrowheads in (B,D,G,I) indicate the positions of fin rays.

**FIGURE 5:**
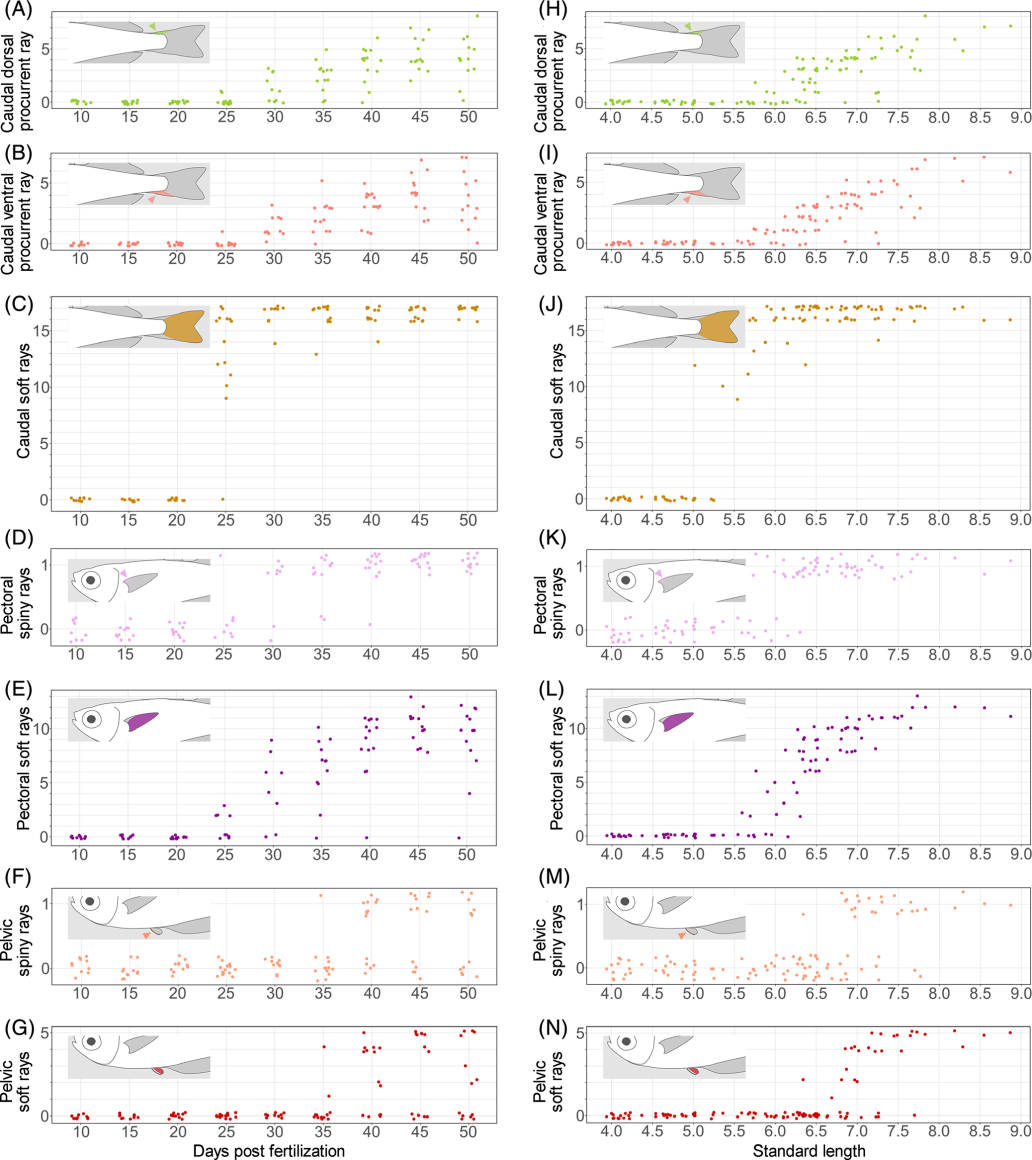
(A–G) Relationships between fin ray (procurrent, spiny, or soft) number and days postfertilization in caudal (A–C), pectoral (D,E), and pelvic (F,G) fins. To reduce data overlap, all points are jittered along *x* and *y* axes. (H–N) Relationships between fin ray number and standard length in caudal (H–J), pectoral (K,L), and pelvic (M,N) fins. To reduce data overlap, all points are jittered along the *y* axis. Schematic drawings in each graph (A–N) indicate the position of each type of fin ray. Arrowheads in (A,B,D,F,H,I,K,M) indicate the positions of fin rays.

Fin development can correlate with absolute age or body size.^15^, ^16^, ^33^ To test the correlation between fin ray numbers and age, we analyzed the data on the increase of fin ray numbers with days postfertilization (Figures 4A–E and 5A–G). Development of the first DF spiny rays was usually apparent after 30 dpf (Figure 4A), while those of the second DF were usually apparent from 30 to 35 dpf (Figure 4B). Development of some second DF soft rays was usually apparent after 25 dpf (Figure 4C). Anal fin spiny rays were usually apparent from 25 to 30 dpf (Figure 4D), and anal fin soft rays usually after 25 dpf (Figure 4E). Some caudal dorsal procurrent rays were usually apparent from 25 to 40 dpf (Figure 5A), and caudal ventral procurrent rays from 25 to 40 dpf (Figure 5B). Development of caudal fin soft rays typically occurred from 20 to 25 dpf (Figure 5C). Pectoral fin spiny rays were usually apparent after 25 dpf (Figure 5D), and soft rays from 25 to 35 dpf (Figure 5D). Pelvic fin spiny and soft rays were usually apparent after 35 dpf (Figure 5F,G, respectively). However, there were some exceptions to this, and fin ray numbers for each day were variable.

To test the correlation between fin ray numbers and body size, we analyzed the data on fin ray number increase with SL (Figures 4F–J and 5H–N). The first DF spiny rays were usually apparent from 6.5 to 7.0 mm SL (Figure 4F), and the second DF spiny rays from 6.0 to 6.5 mm SL (Figure 4G). Second DF soft rays were usually apparent from 6.0 to 7.0 mm SL (Figure 4H). Anal fin spiny rays were usually apparent from 6.0 to 6.5 mm SL (Figure 4I), and soft rays from 5.5 to 6.0 mm SL (Figure 4J). Caudal dorsal procurrent rays were usually apparent from 6.0 to 6.5 mm SL (Figure 5H), caudal ventral procurrent rays from 6.0 to 6.5 mm SL (Figure 5I), and soft rays from 5.5 to 6.0 mm SL (Figure 5J). Pectoral fin spiny rays were usually apparent from 5.5 to 6.5 mm SL (Figure 5K), and soft rays from 5.5 to 6.5 mm SL (Figure 5L). Pelvic fin spiny rays were usually apparent from 7.0 to 7.5 mm SL (Figure 5M), and soft rays from 6.5 to 7.0 mm SL (Figure 5N).

Although our data indicate a correlation between SL and fin ray appearance, exceptions exist. Because dpf and SL correlate weakly with the timing of appearance and number of fin rays on median and paired fins, neither age nor body size is suitable for staging fin development.

### 2.3 DF stage

Development from the embryo to juvenile could be staged using a fin development table. In teleosts, fins containing skeletal supports are established in fold‐like structures.^35^ Therefore, we select specific character states relating to the dorsal area of the LMFF, and the first and second DFs, to define developmental stages.

#### 2.3.1 DF Stage 1

**FIGURE 6:**
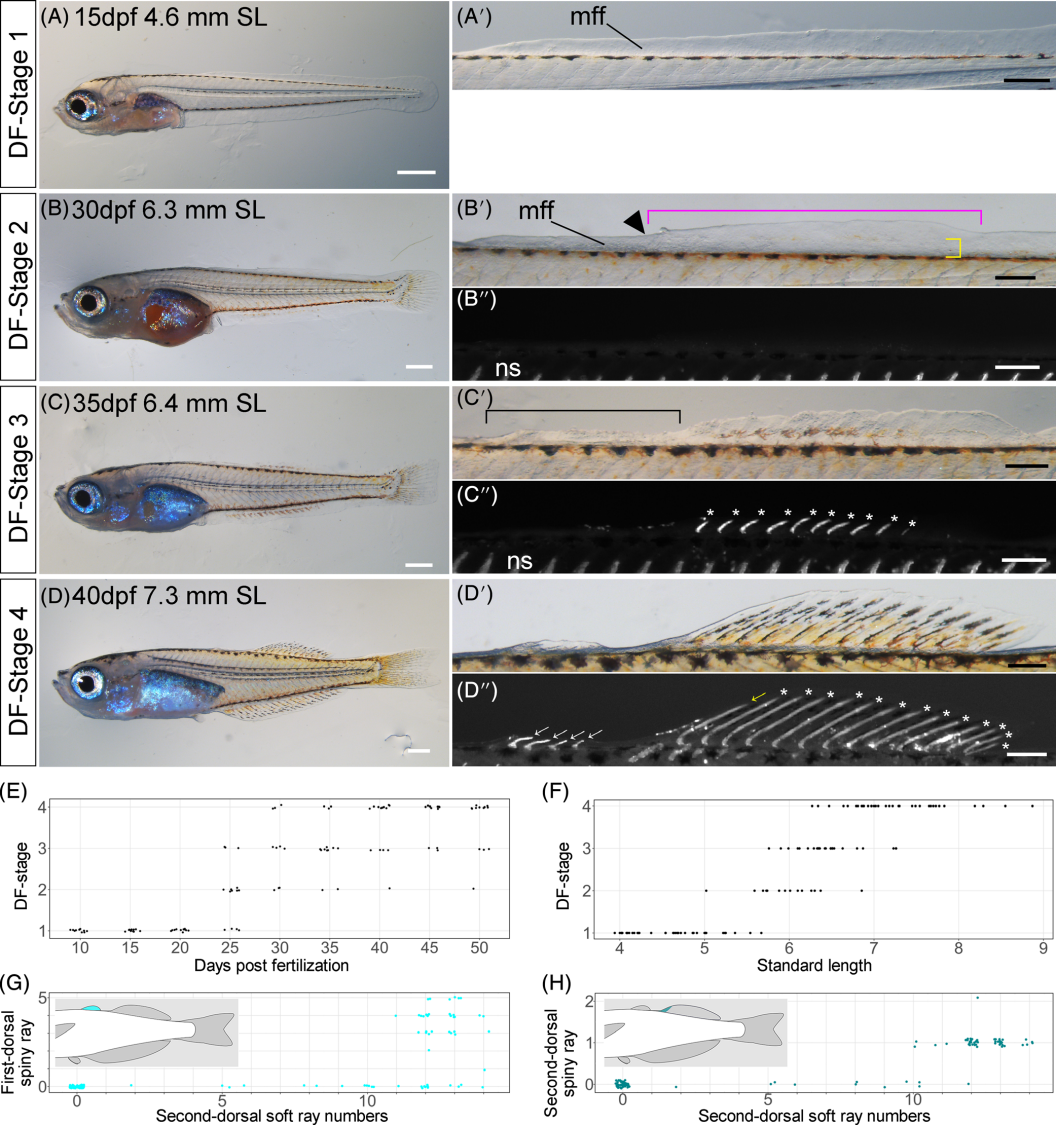
Overview of dorsal fin development in postembryonic stages of *Melanotaenia praecox*. (A–D″). Lateral views of DF Stages: 1 (A,A′), 2 (B–B″), 3 (C–C″), and 4 (D–D″). Magnified views of dorsal fins are shown in (A′,B′,C′,D′; bright field images) and (B,C″,D″; grayscale fluorescent images). mff, median fin fold; ns, neural spine. Black arrowhead shows the difference in level of the dorsal area of the larval median fin fold. Magenta brackets in (B′) indicate presumptive second dorsal fin area. Yellow brackets in (B′) indicate a clump (condensation) of mesenchymal cells. White asterisks indicate soft rays. Black brackets in (C′) indicate future dorsal fin sites. White arrows in (D″) indicate spiny rays. (E–H) Relationships between: (E) days postfertilization and DF stage; (F) standard length and DF‐stage; and (G, H) numbers of second dorsal soft and spiny rays on the first (G) or second (H) dorsal fin. To reduce data overlap, all points in (E,G,H) are jittered along *x* and *y* axes. Schematic drawings in each graph (G,H) indicate the position of each type of fin ray. Scale bars: 500 μm (A–D), and 200 μm (A′,B′,B″,C′,C″,D′,D″).

During early DF development, the fin fold lacks visible bony structure, and the height of the LMFF is almost entirely even (Figure 6A,A′). We regard that period during which a flat, continuous LMFF lacks protrusions or outgrowths as DF Stage 1.

#### 2.3.2 DF Stage 2

With development, outgrowths of the LMFF occur in the presumptive second DF area, in which a clump (condensation) of mesenchymal cells can be observed (yellow bracket, Figure 6B′). Heights of the fin fold differ between anterior and posterior regions (magenta bracket, Figure 6B′, presumptive second dorsal‐fin area). At this stage (DF Stage 2), a difference in level (black arrowhead, Figure 6B′, a gap) of the dorsal area of the LMFF exists, and no first or second DF ray is apparent using either stereo microscopy (Figure 6B′) or fluorescent microscopy when stained with Alizarin Red or calcein (Figure 6B″).

#### 2.3.3 DF Stage 3

With further growth, the second DF soft rays become apparent using both stereo microscopy (Figure 6C′) and fluorescent microscopy when stained with Alizarin Red or calcein (white asterisk, Figure 6C″) as outgrowths of the LMFF (Figure 6C′). At this stage (DF Stage 3) the first DF rays are not apparent, and the future site of the first DF has not outgrown (black bracket, Figure 6C′).

#### 2.3.4 DF Stage 4

The anterior‐most region of the LMFF has started to outgrow, and spiny ray skeletons are apparent in the first DF (DF Stage 4). The first spiny ray is apparent in the second DF (yellow arrow, Figure 6D″), and most soft rays are present (white asterisks, Figure 6D″).

#### 2.3.5 Overview of DF stage

The relationship between dpf and DF stage is shown in Figure 6E. Various DF stages occur at any given dpf. The relationship between SL and DF stages is also imprecise (Figure 6F). Accordingly, we use states of DF development (independent of dpf and SL) to define DF stages.

Spiny rays on the second DF emerge only after 10 soft rays have emerged (Figure 6H), indicating that spiny rays develop later than soft rays. First DF spiny rays first emerge after 11 soft rays have emerged on the second DF (Figure 6G). Because most adults have 11–14 soft rays on the second DF (Figure 2B and Table 1), first DF spiny rays appear to develop after most second DF soft rays have appeared. Therefore, we define DF Stage 3 to be when soft rays develop on the second DF, and DF Stage 4 when spiny rays develop on both the first and second DFs. Furthermore, the first dorsal spiny ray development does not successively occur after the second dorsal soft and spiny rays development, suggesting that the developmental modularity of these fins is independent. Thus, the period during the DF formation can be divided into two stages.

### 2.4 Development of other fins

Because neither absolute age nor SL adequately characterize the number of fin rays on any fin (Figures 4 and 5), we relate the development of fin rays on all fins (unpaired and paired) to DF stages.

#### 2.4.1 Anal fin development

**FIGURE 7:**
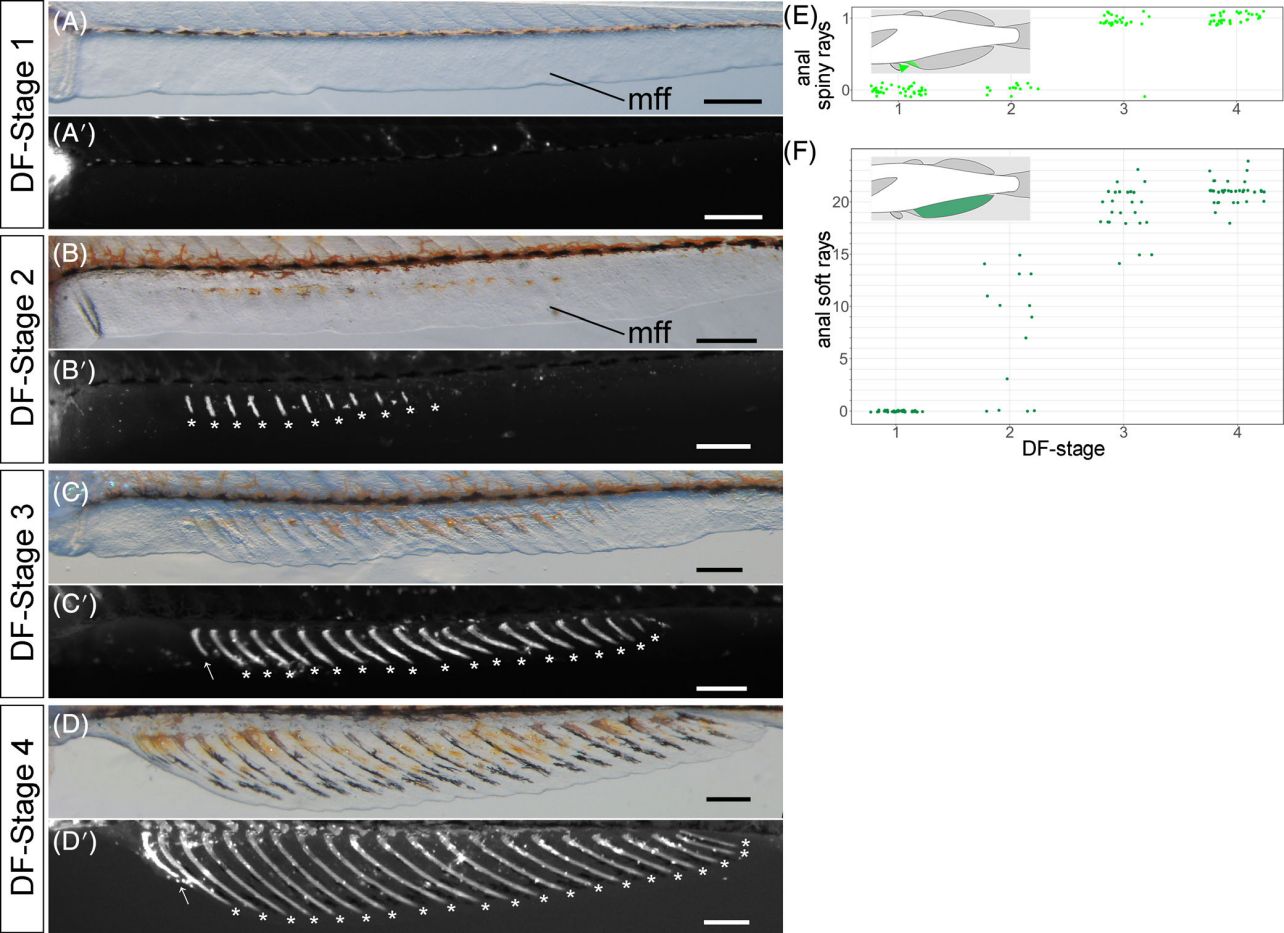
Overview of anal fin development in postembryonic *Melanotaenia praecox* stages. (A–D′) Lateral views of DF Stages: 1 (A,A′), 2 (B,B′), 3 (C,C′), and 4 (D,D′). Bright field (A–D) and grayscale fluorescent (A′–D′) images. mff, median fin fold. Asterisks, soft rays; arrows, spiny rays. (E,F) Relationships between DF stage and number of spiny (E) or soft (F) rays. To reduce data overlap, all points in (E,F) are jittered along *x* and *y* axes. Schematic drawings in each graph (E,F) indicate the position of each type of fin ray. All scale bars: 200 μm.

At DF Stage 1, no individual had an anal fin soft ray, but in some individuals, a clump of mesenchyme was observed within the future site of the anal fin (Figure 7A,A′,E,F); these first emerge at DF Stage 2 (Figure 7B,B′,F; white asterisk, 7B′), and number 0–15; numbers of anal fin soft rays range 14–23 in DF Stage 3 and 18–24 in DF Stage 4. Numbers of soft rays were mostly nonoverlapping between DF Stages 2 and 3. Because the anal fin has 18–22 soft rays in our adult specimen (Figure 1 and Table 1), the anal fin at DF Stage 4 probably has the full complement of these structures. Anal fins of DF Stage 1 and 2 individuals lack spiny rays (Figure 7A,A′,B,B′,E), and all but one DF Stage 3 and 4 individuals had spiny rays (Figure 7C–E; white arrow, 7D′).

#### 2.4.2 Caudal fin development

**FIGURE 8:**
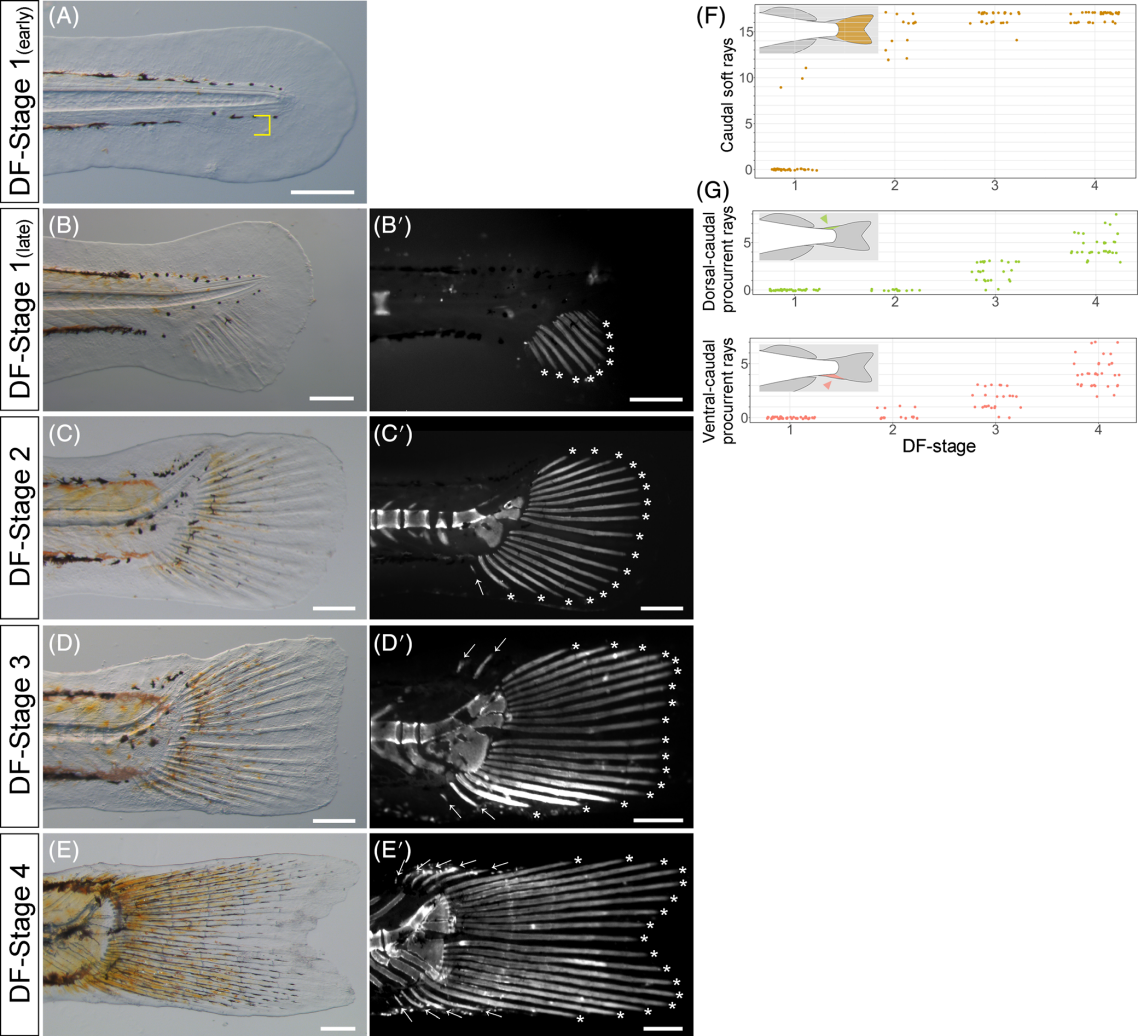
Overview of caudal fin development in postembryonic stages of *Melanotaenia praecox*. (A–E′) Lateral views of early (A) or late (B,B′) phase of DF Stages 1, 2 (C,C′), 3 (D,D′), and 4 (E,E′). Bright field (A–E) and grayscale fluorescent (B′–E′) images. Yellow brackets in (A) indicate a clump (condensation) of mesenchymal cells. Asterisks indicate soft rays; arrows indicate procurrent rays. (F–H) Relationships between DF stage and number of soft rays (F), caudal dorsal procurrent rays (G), and caudal ventral procurrent rays (H). To reduce data overlap, all points in (F–H) are jittered along *x* and *y* axes. Schematic drawings in each graph (F–H) indicate the position of each type of fin ray. All scale bars: 200 μm.

Caudal fin soft rays emerge at DF Stage 1 (Figure 8A–B′,F); all DF Stage 2–4 individuals had caudal soft rays (Figure 8B–F). In early‐phase caudal rays, a clump (condensation) of mesenchyme (yellow bracket, Figure 8A) is apparent within the future site of the caudal fin (Figure 8A). Numbers of caudal fin soft rays at DF Stage 1 range 0–11, and at DF Stage 2, range 12–17 (Figure 8F). Because most DF Stage 3 and 4 individuals had 16 or 17 caudal fin soft rays (Figure 8D–E′,F), and adults 16–18 (Figure 2B and Table 1), the full complement of soft rays is mostly present by DF Stage 3.

The timing of caudal procurrent ray emergence occurs later and increases more gradually than soft rays. Caudal dorsal procurrent rays are absent at DF Stages 1 and 2 (Figure 8A–C′,G), but most DF Stage 3 and 4 individuals had them (Figure 8D–E′,G). Numbers of caudal dorsal procurrent rays at DF Stage 3 range 0–3, and at DF Stage 4, range 3–8. Although caudal ventral procurrent rays were lacking or scarce at DF Stages 1 and 2 (Figure 8A–C′,H; white arrow, Figure 8C′), most DF Stage 3 and 4 individuals had them (Figure 8D–E′,H). Numbers of caudal ventral procurrent rays at DF Stage 3 ranged 0–3, and at DF Stage 4, range 2–7. Numbers of caudal procurrent rays were mostly nonoverlapping between DF Stages 2–4.

#### 2.4.3 Pelvic/pectoral fin development

**FIGURE 9:**
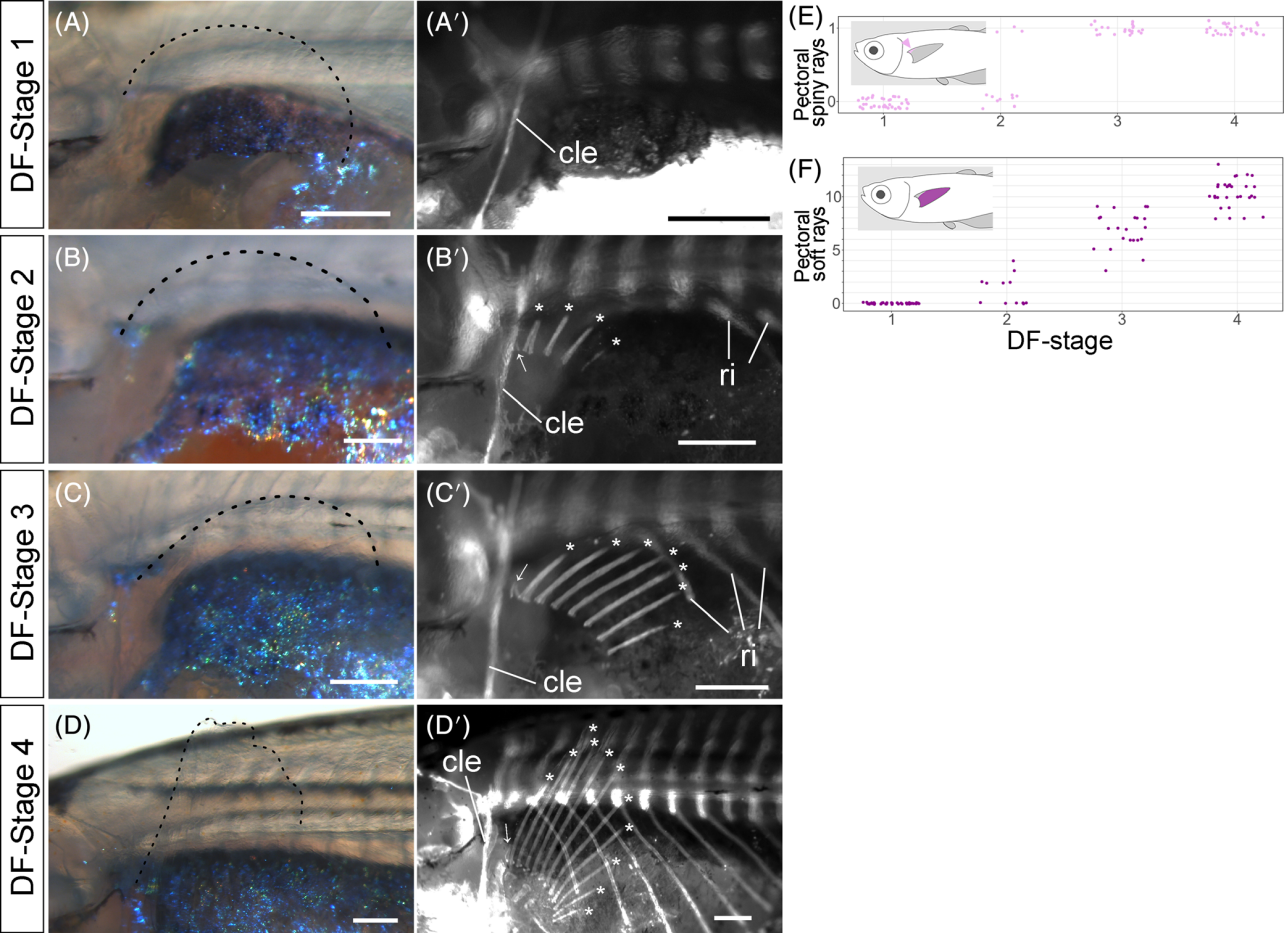
Overview of pectoral fin development in postembryonic stages of *Melanotaenia praecox*. (A–D′) Lateral views of DF Stages: 1 (A,A′), 2 (B,B′), 3 (C,C′), and 4 (D,D′). Bright field (A–D) and grayscale fluorescent (A′–D′) images. cle, cleithrum; ri, rib. Dashed lines, outline of pectoral apical fold or pectoral fins; asterisks, soft rays; arrows, spiny rays. (E,F) Relationships between DF stage and number of spiny (E) or soft (F) rays. To reduce data overlap, all points in (E,F) are jittered along *x* and *y* axes. Schematic drawings in each graph (E,F) indicate the position of each type of fin ray. All scale bars: 200 μm.

Pectoral fin soft and spiny rays first appeared at DF Stage 2 (Figure 9B–F; white asterisk and arrow, Figure 9B–D′). Numbers of pectoral fin soft rays overlap little between stages: DF Stage 2 from 0 to 4; DF Stage 3 from 3 to 9; and DF‐stage 4, from 8 to 13 (Figure 9F). Pectoral fins of most DF Stage 1 and 2 individuals lack spiny rays (Figure 9A,B′,E and 9B′ [an exceptional individual]); all DF Stage 3 and 4 individuals had a pectoral fin spiny ray (Figure 9C–E; white arrow, 9C′,D′).

**FIGURE 10:**
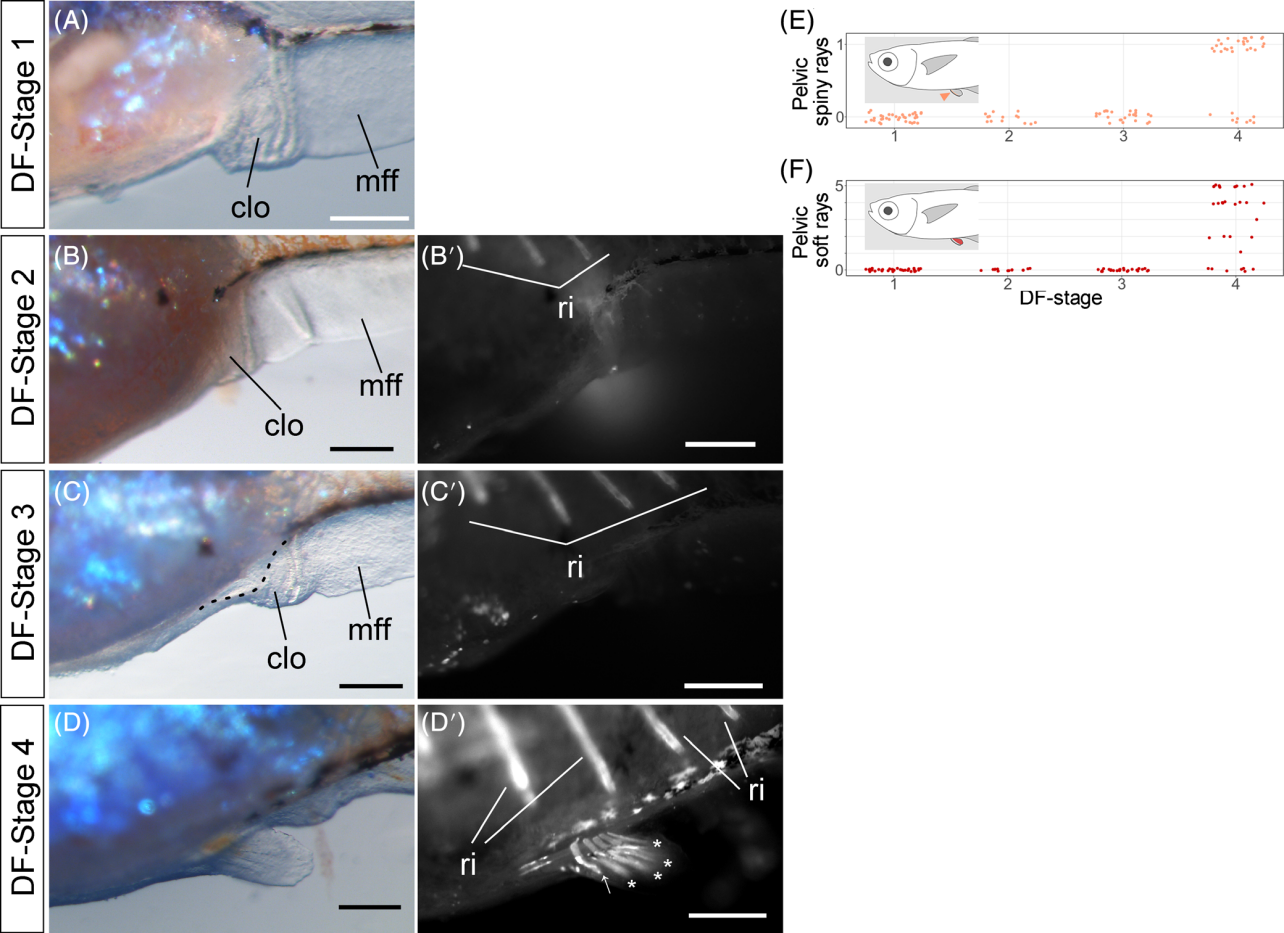
Overview of pelvic fin development in postembryonic *Melanotaenia praecox* stages. (A–D′) Lateral views of DF Stages: 1 (A), 2 (B,B′), 3 (C,C′), and 4 (D,D′). Bright field (A–D) and grayscale fluorescent (B′–D′) images. clo, cloaca; mff, median fin fold; ri, rib. Dashed lines, outline of pelvic fin bud; asterisks, soft ray; arrow, spiny rays. (E,F) Relationships between DF‐stage and number of spiny (E) or soft (F) rays. To reduce data overlap, all points in (E,F) are jittered along *x* and *y* axes. Schematic drawings in each graph (E,F) indicate the position of each type of fin ray. All scale bars: 200 μm.

No DF Stage 1–3 individuals had pelvic fin rays. Soft and spiny rays first emerged at DF Stage 4, and numbered 1–5 (soft rays) and 0 or 1 (spiny rays) (Figure 10D–F; white asterisk and arrow, Figure 10D,D′). Pelvic fin ray development may commence near DF Stage 4, and these are the last to develop on all fins.

## 3 DISCUSSION

### 3.1 Staging fin development in *M. praecox*

**FIGURE 11:**
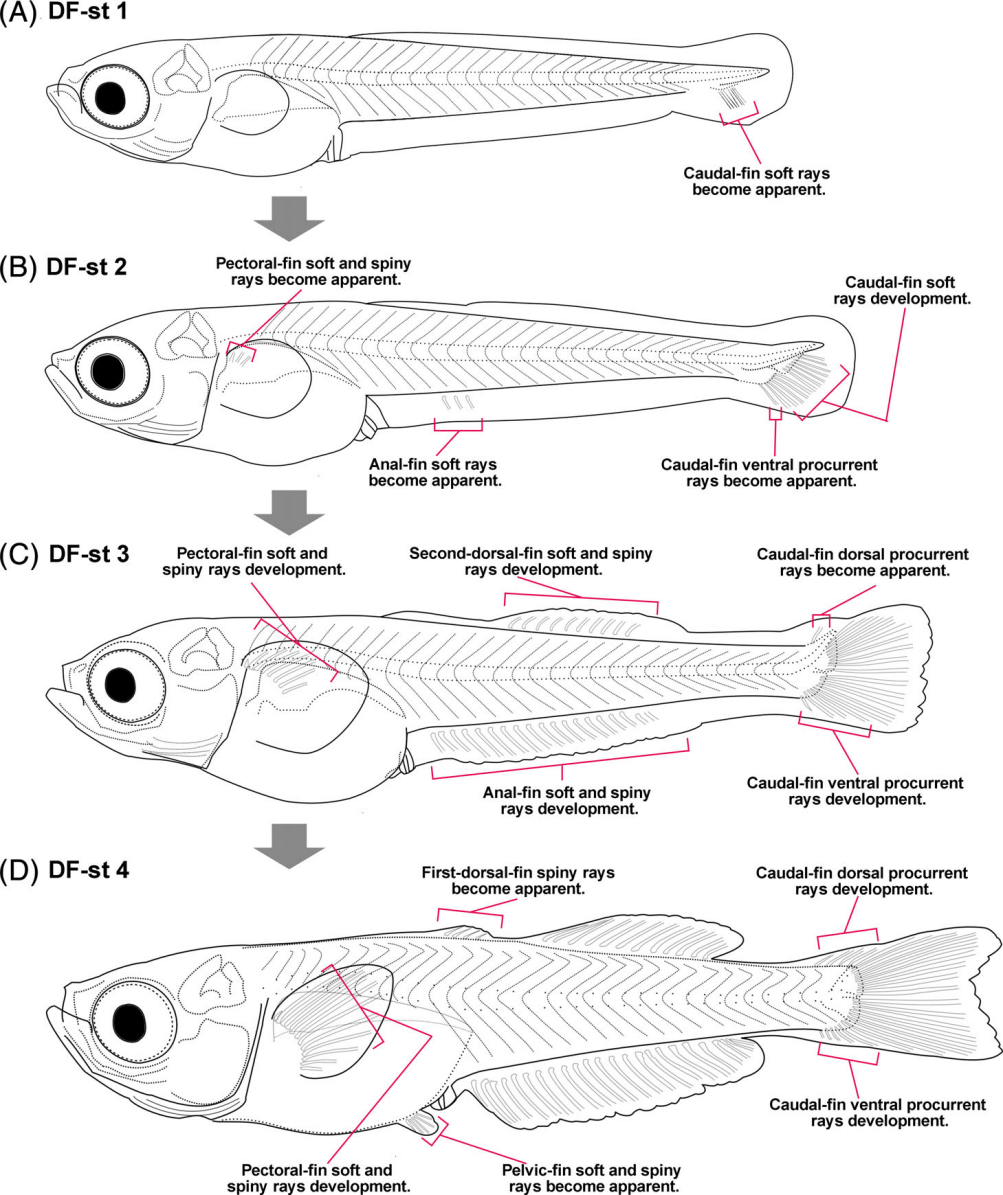
Schematics of *Melanotaenia praecox* DF Stages: (A) 1, (B) 2, (C) 3, and (D) 4.

While fin development in zebrafish correlates well with SL,^33^ this relationship is not well defined in *M. praecox* for either SL or age. Accordingly, for this species, neither is suitable for defining stages of fin development. Fin development of *A. ocellaris* also correlates poorly with age and SL.^16^ Because some features correlate poorly with age and SL, qualitative criteria (e.g., pelvic bud, pigmentation) have been used to stage fish such as goldfish^32^, ^34^ and *A. ocellaris*.^16^ However, to establish meaningful postembryonic stages pertaining to fin development, the morphology of the DF‐fold and DFs suffices. Therefore, we categorize larval development of *M. praecox* into four DF Stages (1–4; Figure 11 and Table 2), each of which is identifiable using a bright field stereo microscope. We describe good correlations between DF stages and fin ray development in all fins (Figures 7, 8, 9, 10). To avoid discrepancy, we recommend using DF stage, for which we considered only DF morphology. However, if further splitting of stages into smaller divisions is required, the morphologies of other fins could be used supplementarily by referring Table 2. Together, we suggest that staging development based on LMFF and fin morphology can facilitate research on fin development in *M. praecox*.

**TABLE 2:** Detailed description of fin ray ontogeny of *Melanotaenia praecox*.

DF stage		Other fins morphology
DF Stage 1	Early phase	Fin ray of all fins are absent
	Late phase	Caudal fin soft rays are present
DF Stage 2	Early phase	Only caudal fin soft rays are present
	Late phase	Pectoral fin soft rays, pectoral fin spiny rays, anal fin soft rays or caudal ventral procurrent rays are present
DF Stage 3	Early phase	Caudal dorsal procurrent rays are absent
	Late phase	Caudal dorsal procurrent rays are present
DF Stage 4	Early phase	Pelvic fin soft and spiny rays are absent
	Late phase	Pelvic fin soft and spiny rays are present

### 3.2 Regulation of fin development

Correlations between DF stages and development of fin rays on each fin indicate that development of each fin ray may be synchronized with DF development and be independent of body size (SL). For example, anal fin spiny rays were absent at DF Stages 1 and 2, but, with one exception, they were present at DF Stages 3 and 4. Teleost metamorphosis typically defines entry into juvenile stages,^16^, ^36^, ^37^ and is triggered by thyroid hormones.^17^, ^37^, ^38^, ^39^ In many fish species, fin ray development also occurs just prior to the larval–juvenile transition.^32^, ^33^, ^34^ In zebrafish, perturbations in thyroid hormone signals affect fin development. For example, chemical inhibition of a thyroid hormone stunted pectoral and pelvic fin growth^40^; in hypothyroid and hyperthyroid conditions, changes in the caudal fin skeleton were consistent with coordinated shifts along the proximodistal axis^41^; and musculoskeletal abnormalities in T3 hyperthyroid cases have been described.^42^ We speculate that fin ray development is controlled by thyroid hormones, and that fin ray development may synchronize independent of SL.

The sequence of *M. praecox* fin formation is similar to that of many species, including zebrafish,^33^ clownfish,^16^ and cichlids.^15^ However, in some acanthomorph fish such as damselfish^43^ and bluefin tuna^44^ the first DF forms before the anal or second DF. Thus, the sequence of fin formation seems not to be highly conserved in fish species. In addition, the directions regarding the order of skeletal development in each fin are not highly conserved.^45^

The three types of fin rays in *M. praecox* (soft, spiny, and procurrent) have been historically classified based on their morphology.^10^ We report that spiny and procurrent rays in each fin emerge after neighboring soft rays first develop (Figures 6, 7, 8, 9, 10, 11), as often reported in the development of other fishes.^16^, ^43^, ^46^ However, spiny ray development precedes soft ray development in some species, such as bluefin tuna.^44^, ^47^, ^48^ In some acanthomorpha taxa, the direction regarding the order of development of the spiny rays is different from that of the soft rays.^45^ Because some expressed genes differ in spiny and soft ray domains,^5^ these structures may behave as independent developmental modules, facilitating morphological diversity of each type of fin ray.

### 3.3 Features of *M. praecox* as a model fish

Acanthomorph fish in previous studies have required large tanks (*A. burtoni*) or seawater systems (*A. ocellaris*) to rear them in. Thus, the conditions required to maintain these acanthomorph model species have limited study of acanthomorph‐specific features. Establishment of a system to maintain *M. praecox* in a laboratory may facilitate research on these fishes, because *M. praecox* (1) is a freshwater species of (2) small size (that requires a system similar to that of zebrafish and medaka), that is (3) easy to breed prolifically throughout the year, with a (4) short generation time. It represents a more easily reared, and potentially better model species to use for research on fish development. Our staging system based on fin development will facilitate such studies. We are also developing a genetic‐engineering system, including targeted mutagenesis and transgenesis, for *M. praecox*.^27^ One disadvantage of using *M. praecox* in fin‐development studies is that spiny rays appear relatively late (from 30 dpf) compared with *A. burtoni* (7–8 dpf) and *A. ocellaris* (6–8 dpf). However, this delayed development has allowed us to report differences in the timing of soft and spiny ray development.

## 4 EXPERIMENTAL PROCEDURES

### 4.1 Animal husbandry and embryo culture

All fish were hatched and maintained in the laboratory in 1 or 3 L tanks, ordinarily at 28°C, pH slightly above 7.0, and beneath a light: dark cycle of 14:10 h. In these conditions *M. praecox* released eggs when adult males and females were held together. To collect many eggs, we established 3 L tanks, each containing a single breeding pair. Adults were fed live brine shrimp once or twice daily. Females released 10–20 eggs over woolen mops suction clamped to the tank glass walls each day; eggs adhered to the mops by their filaments. To avoid being eaten, laid eggs were collected immediately after discovery from the breeding tank using tweezers and held in E3 water.^49^ Hatched larvae were transferred to a 250 mL rearing tank, in which their density was ~1–15 individuals per tank. It was not possible to maintain a set larval density per tank across our experiments, because the numbers of eggs that were laid and could be simultaneously collected was small and variable, and embryos often died. Depending on progeny size, larvae were fed live *Paramecium* at least once every 2 days and/or brine shrimp at least once daily. Removal of dead larvae and excreta was performed as required. After the juvenile stage, progeny were moved to larger tanks, ranging from 250 mL up. We maintained embryos from different clutches that spawned on the same day in the same tanks.

Animal care experimental procedures were conducted in accordance with institutional and national guidelines and regulations, and were approved by the Tohoku University Animal Research Committee (permit numbers 2022LsA‐002‐02, 2020LsLMO‐018‐05). The study was carried out in compliance with ARRIVE guidelines.

### 4.2 Larval observation

We define SL as the distance from the snout to the extremity of the notochord in pre‐flexion larvae, and the distance from the snout to the middle of the caudal peduncle in post‐flexion larvae. The mean of three measurements was used in the analysis of SL. Numbers of fin rays were counted from skeletal‐stained images.

To determine SL and fin ray counts, we sampled from 111 larvae, consisting of 10, 11, 11, 9, 8, 12, 14, 14, and 12 larvae from 10, 15, 20, 25, 30, 35, 40, 45, and 50 dpf, respectively. We did not observe the pectoral and pelvic fin rays of some samples (5 samples of 25 dpf embryo, 2 samples of 30 dpf embryo, and 1 sample of 35 dpf). To observe fin ray development, vital bone staining with 13.9 mM Alizarin Red S (AR) or 0.01% calcein solution at pH 7 was used.^50^, ^51^ Only calcein‐stained images are figured because pigmented cells autofluoresce when AR molecules are excited by the laser. Larval *M. praecox* were immersed in the AR solution for 150 min or the calcein solution for 30 min at room temperature. After bone staining, larvae were washed three times with system water and observed by microscope (Leica M205 FA) and photographed (Leica DFC 360 FX). Images were obtained and analyzed with Leica LAS‐AF, LAS‐X, and Adobe Photoshop CS6. Larvae were anesthetized with 0.025% MS222/E3, placed on a 1% agarose‐gel/E3, transferred immediately to a small case filled with system water, and awakened with water. For bright field imagery, some larvae were photographed beneath the microscope before skeletal staining. Images were obtained and analyzed with Leica LAS‐AF, and analyzed with Adobe Photoshop CS6.

### 4.3 Statistical analysis

All statistical analyses and plotting were performed using the R (https://www.r-project.org/) statistical computing package. Scatter plots, which show the transition of the SL, were generated with the R package ggplot2. The local polynomial regression fit shown in Figure 3A was obtained using the loess method.

## FUNDING INFORMATION

This study was supported by JSPS KAKENHI (grant numbers 22K06232, 20H04854, 22H02627, 21K19202, and 21H05768) and Takeda Science Foundation (grant number 2022036015).

## CONFLICT OF INTEREST STATEMENT

The authors have no conflict of interest to declare.

## Data Availability

All raw data and image files are available upon request from the authors.
